# Correlation of bevacizumab-induced hypertension and outcomes of metastatic colorectal cancer patients treated with bevacizumab: a systematic review and meta-analysis

**DOI:** 10.1186/1477-7819-11-306

**Published:** 2013-11-28

**Authors:** Jun Cai, Hong Ma, Fang Huang, Dichao Zhu, Jianping Bi, Yang Ke, Tao Zhang

**Affiliations:** 1 Tongji Medical College, Cancer Center of Union Hospital, Huazhong University of Science and Technology, Wuhan, Hubei 430022, PR China; 2 Department of Oncology, First Affiliated Hospital of Yangtze University, JingZhou, Hubei 44300, PR China

**Keywords:** Bevacizumab, Hypertension, Metastatic colorectal cancer, Vascular endothelial growth factor

## Abstract

**Background:**

With the wide application of targeted drug therapies, the relevance of prognostic and predictive markers in patient selection has become increasingly important. Bevacizumab is commonly used in combination with chemotherapy in the treatment of metastatic colorectal cancer. However, there are currently no predictive or prognostic biomarkers for bevacizumab. Several clinical studies have evaluated bevacizumab-induced hypertension in patients with metastatic colorectal cancer. This meta-analysis was performed to better determine the association of bevacizumab-induced hypertension with outcome in patients with metastatic colorectal cancer, and to assess whether bevacizumab-induced hypertension can be used as a prognostic factor in these patients.

**Methods:**

We performed a systematic review and meta-analysis on seven published studies to investigate the relationship between hypertension and outcome of patients with metastatic colorectal cancer treated with bevacizumab. Our primary endpoint was progression-free survival (PFS). Secondary endpoints were overall survival (OS) and overall response rate (ORR). Hazard ratios (HRs) for PFS and OS were extracted from each trial, and the log of the relative risk ratio (RR) was estimated for ORR.

**Results:**

The occurrence of bevacizumab-induced hypertension in patients was highly associated with improvements in PFS (HR = 0.57, 95% CI: 0.46–0.72; *P* <0.001), OS (HR = 0.50; 95% CI: 0.37–0.68; *P* <0.001), and ORR (RR = 1.57, 95% CI: 1.07–2.30, *P* <0.05), as compared to patients without hypertension.

**Conclusions:**

Bevacizumab-induced hypertension may represent a prognostic factor in patients with metastatic colorectal cancer.

## Background

Colorectal cancer (CRC) is the fourth most common malignancy, and the second most frequent cause of cancer-related death in the United States, given that as many as 20–25% of patients have already developed metastases at initial diagnosis [1]. Vascular endothelial growth factor (VEGF) is the major factor involved in tumor angiogenesis [2]. It promotes endothelial cell survival, migration, and permeability, and stimulates the growth of blood vessels supplying the tumor. Poor prognosis and an increased relapse rate are often correlated with angiogenesis and increased blood vessel density in the primary tumor. Thus, anti-angiogenesis is a major topic of current research.

The VEGF signaling pathway is a target for cancer therapy. A recombinant humanized monoclonal antibody against VEGF, bevacizumab, has been developed to treat metastatic CRC (mCRC), breast cancer, non-squamous non-small cell lung cancer, renal cell carcinoma, ovarian cancer, glioblastoma, and metastatic melanoma [3-11]. Treatment with bevacizumab, however, is associated with various adverse reactions such as gastrointestinal perforations, wound healing complications, hemorrhage, arterial thrombotic events, infection, proteinuria, and hypertension. Nevertheless, the benefits of bevacizumab treatment may still outweigh potential adverse events [12]. Furthermore, bevacizumab has been demonstrated to be relatively safe in association with either irinotecan [13] or oxaliplatin-containing chemotherapy regimens [14], while its specific toxicity profile appears manageable by applying appropriate clinical selection criteria [15].

As mentioned above, arterial hypertension is a common side effect of bevacizumab treatment usually easily managed by standard anti-hypertensive therapy. Interestingly, many clinical trials have found that patients with mCRC treated with bevacizumab who developed hypertension had a better prognosis than those without hypertension [16-22]. These results were obtained through retrospective analysis of a relatively small dataset, but the findings are statistically significant and supported by other studies [6]. Throughout the course of treatment for mCRC, hypertension severity can be evaluated objectively and thus may be useful when making an early decision on whether to alter the course of disease treatment. The potential advantages of such a predictor include the ability to estimate the efficacy and activity of anti-VEGF agents in patients with mCRC.

Thus, the purpose of this study was to perform a systematic review and conduct a meta-analysis to determine if the occurrence of hypertension is a prognostic factor of response and survival for bevacizumab treatment in patients with mCRC.

## Methods

### Data sources

The study was performed using a pre-specified search strategy with a strict eligibility criteria. We did an extensive search of PubMed to retrieve relevant literature that reported the predictive value of hypertension regarding response and/or progression and/or survival in mCRC patients treated with bevacizumab. The search end date was January 2013, with no specified start date. Search term combinations were “b*evacizumab*”, “*avastin*”, and “*hypertension*” in all fields. There were no limits for language, methodological characteristics, or year of publication. All reference lists from the relevant articles and reviews were also examined for additional eligible studies. This study is approved by the Ethic Commity of Cancer Center of Union Hospital. And written informed consent was obtained from the patient for the publication of this report and any accompanying images.

### Selection of studies

Two reviewers (JC, HM) independently carried out a literature search and examined the relevant studies for further assessment. The reference lists of all traced articles were examined manually. Citations selected from this initial search were subsequently screened for eligibility using the following criteria: i) patients with mCRC; ii) combined chemotherapy with bevacizumab, irrespective of chemotherapy used; iii) studies involving the use of other targeted agents were excluded to avoid bias related to drug interactions; iv) curative effect comparison between bevacizumab-induced hypertension arm with no hypertension arm; v) data available for analysis including the incidence of hypertension and sample size.

### Primary and secondary outcomes

The primary outcome was progression-free survival (PFS), defined as the time between randomization and any progression or death from any cause, in relation to the severity of hypertension in patients treated with bevacizumab. Secondary endpoints were overall survival (OS), the time between randomization and any death, and overall response rate (ORR), the sum of partial and complete response rates according to the Response Evaluation Criteria in Solid Tumors [23] with hypertension occurrence as a predictor. Hypertension was graded according to the National Cancer Institute Common Terminology Criteria [24] for Adverse Events (version 3.0, 2003). Grade 1 toxicity is defined as an asymptomatic, transient increase (<24 h) greater than 20 mmHg diastolic or to greater than 150/100 mmHg. Grade 2 is recurrent or persistent (>24 h) or a symptomatic increase greater than 20 mmHg diastolic or to greater than 150/100 mmHg. Grade 3 is hypertension requiring therapy or more intensive therapy than previously provided. Grade 4 is a hypertensive crisis. Outcomes or responses were evaluated by either a comparison between no-hypertension (G0) and all grades of hypertension (G1–4), or a comparison between low-grade hypertension (G0–1) and high-grade hypertension (G2–4), depending on the data available.

### Data extraction

Two reviewers (JC, HM) retrieved data independently and reached a consensus on all examined items. The following information was retrieved: first author, year of publication, number of patients, number of patients eligible for response, and median OS, PFS, ORR, and hazard ratio (HR). For trials included in this meta-analysis, if the log HR and its variance were not explicitly presented, the methods reported by Parmar et al. [25] were used to extract estimates of these statistics. In the case of any disagreement between the two reviewers, a third reviewer (DCZ) would review the data, and the results were attained by consensus. We contacted the authors of trials for the missing data when necessary. Data of study characteristics (concurrent treatment, number of patients, bevacizumab dose, and publication time) and clinical endpoints (PFS, OS, ORR) were then retrieved.

### Data analysis and statistical methods

We calculated relative risk ratios (RRs) and confidence interval (CI) for ORR relating to hypertension severity in patients with bevacizumab-induced hypertension versus controls in the same trial. If the study reported HRs for survival in patients with G0 vs. G1 or higher-grade hypertension, then the comparison was made for the higher grade of hypertension (for example G0 vs. G2 or G3 [or G3–4]). Otherwise, if no other subgroups were reported, the comparison was performed for G0 vs. G1–4 hypertension. HRs were extracted from each trial for PFS and OS, and the log of relative RR was estimated for ORR, and 95% CIs were derived. The HR of each study was either directly collected from the original article, or calculated as suggested by Parmar [25] and Tierney [26]. The number of events (ORRs) was extracted from each study or calculated from the percentages provided.

A meta-analysis of both RRs and HRs was performed, and both fixed-effect and random-effect models were considered depending on the heterogeneity of the included studies. Statistical heterogeneity among trials included in the meta-analysis was assessed by using the Cochran Q statistic, and inconsistency was quantified with the I^2^ statistic that estimates the percentage of total variation across studies due to heterogeneity rather than chance [27]. When substantial heterogeneity was not observed, the pooled estimate was calculated based on the fixed-effects model using the inverse variance method. Otherwise, the pooled estimate was calculated based on the random-effects model using the DerSimonian and Laird method [28].

Publication bias was evaluated using funnel plots for RR (plots of study results against precision), and with the Begg’s [29] and Egger’s [30] tests. Additionally, sensitivity analyses were performed to assess the influence of each study on overall estimate for RR by sequential removal of individual studies. A HR of less than one and a RR value of more than one meant a benefit for patients with bevacizumab-induced hypertension. A two-tailed *P* value <0.05 was considered statistically significant. All statistical analyses were performed using STATA version 11.0 software (STATA, College Station, TX, USA).

## Results

**Figure 1:**
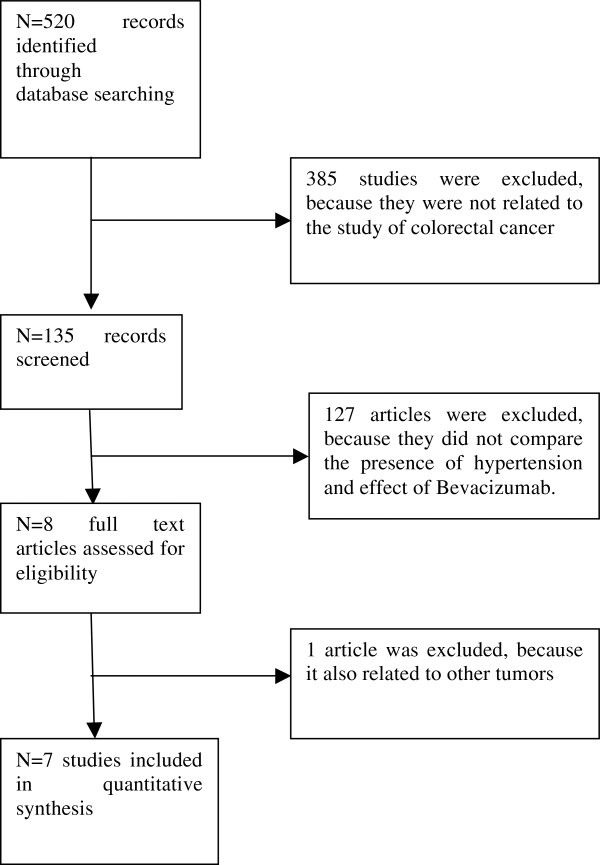
Study flow diagram.

There were 520 publications retrieved from the PubMed search. Among them, seven met the inclusion criteria for this review. The study flow diagram is shown in Figure 1. The main characteristics of the included articles (author/year, reference, line of treatment, bevacizumab dose, number of patients, PFS, OS, ORR) are presented in Table 1. Patients were enrolled according to pre-specified eligibility criteria for each trial. Data regarding the predictive role of hypertension for PFS were available for all seven studies. Secondary outcome data, i.e., OS and ORR, were available for five studies, respectively.

**Table 1:** Characteristics of the seven selected studies

**Author/year [Ref.]**	**Line of treatment**	**Bevacizumab dose**	**No. of patients**	**Median PFS (m)**	**Median OS (m)**	**ORR (%)**
				**HTN vs. No HTN**	**HTN vs. No HTN**	**HTN vs. No HTN**
Scartozzi M/2009 [20]	First-line	5 mg/kg/2w	39	14.5 vs. 3.1	NA vs. 15.1	75% vs. 32%
Rebekah/2009 [21]	First-line	NA	52	NA vs. NA	NA vs. NA	NA vs. NA
De Stefano/2011 [19]	First-line	5 mg/kg/2w or 7.5 mg/kg/3w	74	15.1 vs. 8.3	35.5 vs. 26.7	84.6% vs. 42.6%
Osterlund P/2011 [17]	First- or second-line	5 mg/kg/2w	101	10.5 vs. 5.3	25.8 vs. 11.7	52.6% vs. 45.5%
Horinouchi Y/2011 [18]	First-line	NA	36	16.25 vs. 10	NA vs. NA	60% vs. 23.1%
Dewdney A/2011 [22]	First-line	7.5 mg/kg/3w	45	NA vs. NA	NA vs. NA	71% vs. 78%
Tahover E/2013 [16]	First- or second-line	2. 5 mg/kg/w	181	29.9 vs. 17.2	NA vs. 36.8	NA vs. NA

ORR: Overall response rate; NA: Information not available; PFS: Progression-free survival; OS: Overall survival; HTN: Hypertension group; N0 HTN: No hypertension.

### Efficacy

#### Median PFS

**Figure 2:**
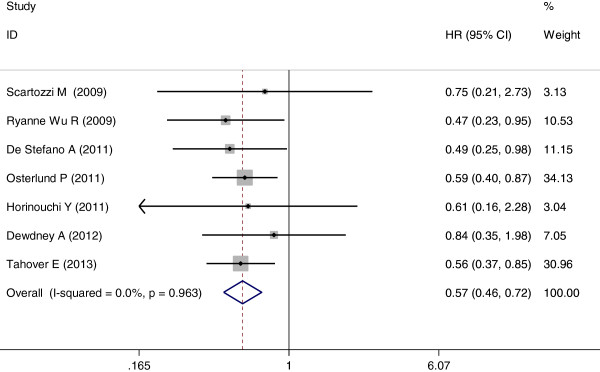
Forest plot for meta-analysis of hypertension occurrence and progression-free survival.

The occurrence of hypertension induced by bevacizumab resulted in a statistically significant improvement in PFS compared with no hypertension (HR = 0.57; 95% CI: 0.46–0.72, *P* <0.001; heterogeneity χ^2^ = 1.45, *P* for heterogeneity = 0.963; I^2^ = 0.0%) (Figure 2). There was no heterogeneity between trials.

#### Median OS

**Figure 3:**
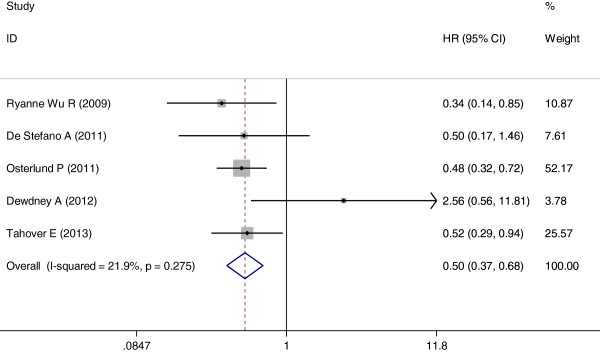
Forest plot for meta-analysis of hypertension occurrence and overall survival.

Among the seven trials selected, five [16,17,19,21,22] included relevant data. The pooled analysis showed that the occurrence of hypertension induced by bevacizumab also resulted in a statistically significant improvement in OS compared with no hypertension (HR = 0.50; 95% CI: 0.37–0.68, *P* <0.001; heterogeneity χ^2^ = 5.12, *P* for heterogeneity = 0.275; I^2^ = 21.9%) (Figure 3). Once again, there was no heterogeneity between trials.

#### ORR

**Figure 4:**
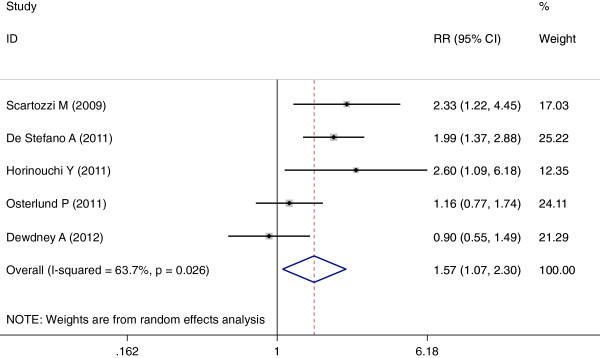
Forest plot for meta-analysis of hypertension occurrence and risk ratio.

**Figure 5:**
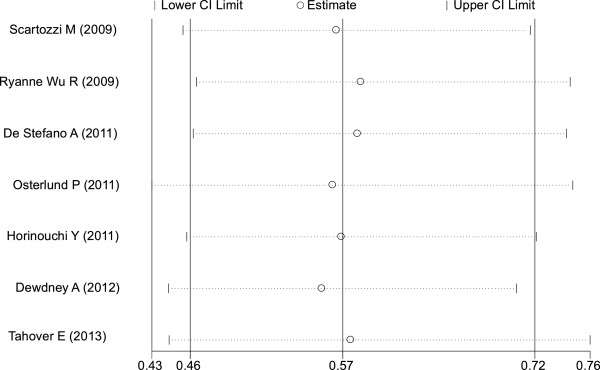
Funnel plot for progression-free survival meta-analysis.

**Figure 6:**
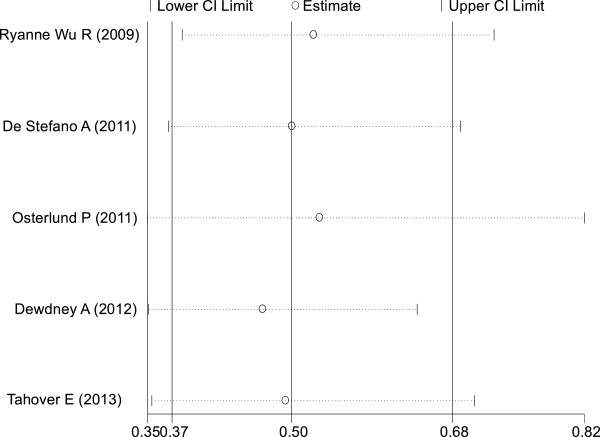
Funnel plot for overall survival meta-analysis.

**Figure 7:**
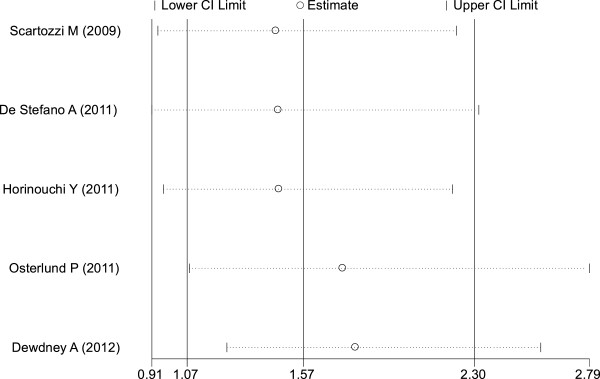
Funnel plot for overall response rate meta-analysis.

Two studies [16,21] did not access this outcome, and were thus excluded from the analysis. The remaining five studies [17-20,22] contained pertinent data. Analysis indicated hypertension induced by bevacizumab was associated with an increase in ORR (RR = 1.57, 95% CI: 1.07–2.30, *P* <0.05) (Figure 4). Because heterogeneity was significant between trials (I^2^ = 63.7%, *P* = 0.026), a combined effects model was used. Funnel plots and the Egger’s test were used to assess publication bias. As reflected in Figures 5, 6, and 7, the shape of the funnel plots appeared symmetrical.

## Discussion

Bevacizumab is widely used as a standard treatment for mCRC; the combined treatment of chemotherapy and bevacizumab has significantly increased the PFS and OS in patients with mCRC. Arterial hypertension is the most common side effect of bevacizumab plus chemotherapy treatment, with an overall incidence of 22–32%, and grade 3/4 events in 11–16% of patients [31,32]. While the hypertension-causing mechanism of bevacizumab is unclear, it is fortunate that bevacizumab-induced hypertension rarely induces severe or life-threatening outcomes. To date, no specific predictive or prognostic biomarkers for bevacizumab treatment have been identified. Some studies have suggested that bevacizumab-induced hypertension could represent a valuable prognostic factor of clinical outcome in advanced-stage CRC patients [16-21]. Thus, it would be interesting to see if hypertension could be a predictive factor in patients with mCRC.

To our knowledge, this is the first meta-analysis that systematically evaluates the correlation of hypertension with survival and response in mCRC patients treated with bevacizumab. Our results indeed demonstrate that bevacizumab-induced hypertension in mCRC patients is significantly associated with PFS and OS. Also, our meta-analysis indicate that the occurrence of hypertension induced by bevacizumab is associated with a statistically significant improvement in ORR, in line with previous studies [19,20].

An outstanding benefit of our study is that patients who are more suitable to bevacizumab treatment could eventually be screened and selected for targeted therapy. Furthermore, it would be of extreme benefit to the fight against cancer if these results were comparable with the outcomes of anti-EGFR monoclonal antibody and KRAS status in CRC [33,34].

## Conclusions

Caution is certainly needed before we may conclude that bevacizumab-induced hypertension is a reliable bio/clinical marker for early screening and diagnosis of patients with mCRC due to the limitation on available data and the relatively small sample size of our study. However, our results should undoubtedly lead to larger sample size, multiple-center clinical studies as well as analyses to further elucidate the correlation between bevacizumab-induced hypertension and mCRC. Thus, feasible and efficient methods to diagnose and treat patients with mCRC at earliest possible stages could be developed.

## Abbreviations

CRC: Colorectal cancer; CI: Confidence interval; HR: Hazard ratio; mCRC: Metastatic CRC; ORR: Overall response rate; OS: Overall survival; PFS: Progression-free survival; RR: Risk ratio; VEGF: Vascular endothelial growth factor.

## Competing interests

The authors report no conflicts of interest. The authors alone are responsible for the content and writing of the paper.

## Authors’ contributions

JC and HM are co-first authors. All authors read and approved the final manuscript.
